# Close Relations, Practitioners or Social Networks: What Affects the Selection of Infant Formula Products?

**DOI:** 10.3390/nu16183089

**Published:** 2024-09-13

**Authors:** Elad Harison, Yael Lahav

**Affiliations:** 1 School of Industrial Engineering and Management, Shenkar College of Engineering and Design, Ramat Gan 5290002, Israel; yaellahav@shenkar.ac.il; 2 Department of Management, Bar Ilan University, Ramat Gan 5290002, Israel

**Keywords:** infant nutrition, infant formula, feeding, decision-making, social circle, medical professionals, social networks, mothers

## Abstract

This study examines which factors influence the preferences of mothers seeking advice on the use of infant formula in feeding practices. The effects of their close social circle, medical professionals and social network forums on feeding choices were evaluated. Data were collected from 638 questionnaires from mothers and were statistically analyzed. Our findings suggest that age may influence the preference to seek advice from a close social circle over consulting with medical professionals (*p*-value = 0.019 < 0.05). The educational level and the number of children impact the preferences of using infant formula over combining it with breastfeeding (*p*-value = 0.000 < 0.05 and *p*-value = 0.004 < 0.05, respectively). The research contributes to the understanding of the complex interplay between various demographic and socioeconomic factors and the decision-making process of mothers based on multiple social and digital sources of influence. The research presents valuable insights for healthcare policymakers and professionals in supporting mothers and providing them with up-to-date information. Feeding information can be distributed through all the communication channels that were examined in this study to benefit different socio-economic groups and to contribute to the well-being of infants in the long-run.

## 1. Introduction

The supply of proper nutrition to infants is pivotal for their growth and long-term health. Feeding infants at birth is based either on breastfeeding or on infant formulas that are usually produced from the milk of animals [1]. Despite the major advances in developing and producing infant formulas, breastfeeding is the best nutritional source for infants. Breastfeeding reduces the risk for multiple health problems, including gastrointestinal and respiratory tract infections, communication problems, allergies and crib death syndrome [2]. Research shows long-term benefits, including reduced risk of obesity and diabetes and increased childhood and adolescent intelligence, that are associated with breastfeeding in infancy [3,4]. Mothers who choose to breastfeed their babies reduce postnatal bleeding and return to pre-pregnancy weight in shorter periods compared to non-breastfeeding women. Breastfeeding women also have a lower likelihood of suffering from breast cancer, ovarian cancer and osteoporosis in the long term [5,6,7]. Consequently, in recent years, the World Health Organization (WHO) declared unprecedented worldwide campaigns that were pro-breastfeeding and against the broad use of infant formulas, including the promotion of bans on advertising these products and the addition of warning labels to them to ensure that breastfeeding replacements were used by mothers only as a last resort. The WHO set a target to increase by 2025 worldwide breastfeeding rates by more than 50%. Though the WHO’s recommendations largely affect health policy guidelines and governments worldwide invest substantial efforts in implementing them, the percentage of women who do not breastfeed their babies remains relatively high [8,9,10].

Beyond the primary decision whether to breastfeed or to use an infant formula, another significant decision is the type of formula given to babies. The choice of infant formula and the feeding regimen of the baby are critical, as inappropriate feeding habits or a defect formula cause significant cognitive and physiological damages that are irreversible. Moreover, infants cannot complain or indicate the lack of ingredients essential for their development. In the year 2003, Remedia, an Israeli company specializing in infant formulas, began offering a new soy-based formula, which was manufactured by the German company Humana. In October 2003, several infants were hospitalized with symptoms of apathy and convulsions. The common cause of their illnesses, to be identified only a month later, in November 2003, was consuming the new formula. It was later discovered that the new formula contained an insufficient quantity of vitamin B1, an essential vitamin for newborns, causing the death of four infants and various long-term motorial, neurological and cognitive damages to more than sixty others [11,12] (it is important to note that lessons from the Remedia case and other cases affected infant formula safety regulations to prevent similar occurrences).

Therefore, choosing the right infant formula is not only challenging but also, to a large extent, a question of life and death. In this respect, parents often seek advice and information that are likely to influence their decisions and consumer behavior patterns. Family members, professionals (such as clinic nurses, pharmacy workers and doctors) and online sources (including social networks and online maternal forums) are among the frequently used channels of communication for mothers [13]. However, it is important to mention that whatever decisions are taken in the period before and after the birth, many women may decide to add formula or to stop breastfeeding after the delivery despite their initial planning to breastfeed, to rely on infant formula or to combine both [14].

This paper aims to explore how different social and personal attributes of mothers affect their choices to seek advice regarding the selection of infant formula and feeding regimens for their babies from their close social circle, professionals or from social media. Section 2 of this paper presents the literature review and includes the research hypotheses. Section 3 and Section 4 describe the methodology and the results of the study. Finally, the discussion is brought up in Section 5. Conclusions, implications of the study and limitations are presented in Section 6.

## 2. Literature Review

Our study examines how parents choose a specific infant formula and a feeding regimen for their babies. There are several stages to infant feeding decisions: whether to breastfeed, when and whether to add formula and when to stop breastfeeding.

Several studies explore the factors that affect the decision to breastfeed [15,16], while Ballesta-Castillejos et al. (2020) [17] found five factors related with the decision of mothers to breastfeed: partner support, previous experience in breastfeeding, having two or more children, attending breastfeeding education and having medical issues during pregnancy. Matriano et al. (2022) [14] explore the factors that influence women’s decisions on infant feeding from the period before birth to throughout the breastfeeding continuum. Moreover, many women change their initial decision after delivery due to challenges that they experienced with breastfeeding. Another main reason is returning to work, which limits their ability to breastfeed. The decision of selecting an infant formula is often based on brand recognition, the formula that was provided at the hospital, previous experience or recommendations, mostly by friends, family or health professionals [13]. Four categories were found as influencers of the decisions of mothers to use or to avoid infant formulas: nutritional benefits for babies in each method of feeding, maternal benefits, knowledge about infant feeding, and personal and professional support. Additionally, marital status, education, age, culture and job security may affect women’s decisions to breastfeed their babies or to use infant formulas [18,19,20].

We assume that the likelihood of preferring advice from the close social circle over medical professionals may be affected by multiple variables as defined by the following hypothesis:

**Hypothesis** **1:** *(a) The older the mother is, the more likely that she would prefer advice from her close social circle over medical advice.*

*(b) A more educated mother is likely to prefer advice from her close social circle over medical advice.*

*(c) The higher salary that the mother receives, the more likely that she would prefer advice from her close social circle over medical advice.*

*(d) Mothers living in a central location are more likely to prefer advice from their close social circle over medical advice than mothers living in peripheral locations.*

*(e) Mothers with more than one child are more likely to prefer advice from their close social circle over medical advice than mothers who gave birth to their first child.*

The effects of social networks (and primarily Facebook) on customer behavior, in particular in the domain of medical and para-medical decision-making, have been broadly studied (see for example [21,22,23]). Different studies highlight the use of social networks as a platform for supporting breastfeeding mothers [24,25,26]. In particular, the role of social networks was primarily examined as a source of information and support in addition to consulting medical professionals. However, the use of social networks as a primary or an additional source of information does not come without risks. Social network users utilize both the relative anonymity and the trust of other users, either to promote suboptimal infant formulas for large groups of mothers (whether these products match the needs of their babies or not) or to post misleading recommendations due to malicious intentions [27,28,29]. Therefore, the question of whether mothers would prefer to consult with their peers on social networks over consulting with medical professionals arises (i.e., preferring crowd wisdom over medical know-how). In this respect, it is interesting to examine whether social network posts substitute, rather than complete, medical opinions. Following this line of argumentation, the following hypothesis is constructed:

**Hypothesis** **2:** *(a) The older the mother is, the more likely that she would prefer advice from social network forums over medical advice.*

*(b) A more educated mother is likely to prefer advice from social network forums over medical advice.*

*(c) The higher salary that the mother receives, the more likely that she would prefer advice from social network forums over medical advice.*

*(d) Mothers living in central locations are more likely to prefer advice from social network forums over medical advice than mothers living in peripheral locations.*

*(e) Mothers with more than one child are more likely to prefer advice from social network forums over medical advice than mothers who gave birth to their first child.*

Multiple studies found that working mothers prefer using infant formula over breastfeeding [30,31]. This decision is motivated either by the need to preserve a continuous stream of income (as is the case for mothers in developing countries) [32,33] or by the motivation of professionals to ensure the ongoing development of their career path, despite the need to take maternal leave and dedicate time to feeding their baby [34,35]. Following the review studies on the different attributes of mothers and their decisions regarding the choice of infant formula for their babies, the following hypotheses are brought herein:

**Hypothesis** **3:** *(a) The older the mother is, the higher the percentage of meals that combine infant formula in the first three months.*

*(b) The higher the mother’s education, the higher the percentage of meals that combine infant formula in the first three months.*

*(c) The higher salary that the mother receives, the higher the percentage of meals that combine infant formula in the first three months.*

*(d) The percentage of meals that combine infant formula in the first three months is higher among mothers living in central locations in comparison to mothers living in peripheral locations.*

*(e) The percentage of meals that combine infant formula in the first three months is higher among mothers with more than one child in comparison to mothers who gave birth to their first child.*

**Hypothesis** **4:** *(a) The older the mother is, the more likely that she would prefer the use of infant formula over combining it with breastfeeding.*

*(b) A more educated mother is likely to prefer the use of infant formula over combining it with breastfeeding.*

*(c) Mothers living in central locations are more likely to prefer the use of infant formula over combining it with breastfeeding than mothers living in peripheral locations.*

*(d) Mothers with more than one child are more likely to prefer the use of infant formula over combining it with breastfeeding than mothers who gave birth to their first child.*

Since their introduction online, social networks and online discussion forums have become popular loci for mothers seeking information about the use of infant formulas. For instance, questions raised by users of these forums include their properties as nutritional alternatives and the extent to which they can complement or replace breastfeeding practices [36]. Morse and Brown (2021) [25] highlight the role of social networks as media that provide new and young mothers confidence about their feeding choices, beyond functioning as knowledge depositories for them. However, the World Health Organization (2022) indicates that corporations utilize social networks for aggressive marketing and promotion of their infant formulas, overlooking the particular needs of both the mothers and babies that are described in online posts. This report raises concerns about the validity of these forums as an objective source of information. Therefore, young and new mothers seeking advice may choose to address other experienced mothers within their close social circle as a preferable alternative. Following the findings of these studies, Hypothesis 5 is constructed as follows:

**Hypothesis** **5:** *Infant formula feeding selections, meal preferences, and the choice of mothers to apply to their close social circle and to a social network for advice are correlated.*

## 3. Materials and Methods

This study is based on a survey designed to investigate the factors influencing mothers’ decisions regarding the use of infant formula and the sources of advice they rely on. The survey was conducted across various regions, targeting mothers with children aged 0–12 months. The questionnaire was developed to capture a wide range of variables, including demographic information, feeding practices and the preferred sources of advice on infant formula use. The survey was distributed both online and in person to ensure a diverse and representative sample of respondents.

A statistical analysis of data collected via questionnaires that were distributed to Israeli mothers through online forums in social networks and WhatsApp groups between May 2023 and June 2023 was carried out. In total, questionnaires from 638 mothers were collected. The questionnaires were examined for completeness of the responses, and none of the questionnaires were excluded. The statistical analysis and testing of the hypotheses then followed and were carried out by using Python and its statistical libraries (numpy and scipy.stats).

The questions were designed to measure both the dependent and independent variables. The dependent variables focused on the mother’s preference for advice from her close social circle over medical advice (denoted as MOMS_OVER_MED) and her preference for advice from online social network forums over medical advice (denoted as NETWORKS_OVER_MED). The independent variables included the mother’s age (AGE), education level (EDUCATION), income (INCOME), geographical location (LOCATION, indicating whether the mother lives in the periphery or center), the number of children (CHILDREN) and whether the mother combines breastfeeding with infant formula (NUTRITION).

The statistical analysis used multinomial logistic regression models. This method was chosen due to the categorical nature of the dependent variables, which include multiple levels of preference. The models were used to determine the significance of the independent variables in predicting the outcomes of the dependent variables. Additionally, the multinomial logistic regression model was used to teste the combined effect of all independent variables on MOMS_OVER_MED. Another analysis examined the influence of EDUCATION and CHILDREN on the mother’s preference for infant formula over a combined feeding regimen (MEAL) by using Goodness-of-Fit statistics and parameter estimates to interpret the odds ratios associated with each independent variable. Chi-Square tests were applied to test the relationships between NUTRITION and MEAL, as well as between the preferences for medical advice and advice from social networks. Subsequent multinomial logistic regression was applied to explore the interaction between MOMS_OVER_MED and NETWORKS_OVER_MED. All statistical analyses were performed using a significance level of *p* < 0.05.

## 4. Results

The study examines five hypotheses related to a mother’s choice between medical advice, advice from her close social circle and advice from online social network forums on the use of infant formula. The statistical analysis utilizes data collected from a survey. Among the major dependent variables examined are the preference to seek advice from a close social circle over medical advice (MOMS_OVER_MED) and the preference to seek advice from a social network forum over medical advice (NETWORKS_OVER_MED). The independent variables include the mother’s age (AGE), education level (EDUCATION), income (INCOME), whether the mother lives in the periphery or in the center (LOCATION), the number of children (CHILDREN) and whether the mother combines breastfeeding with infant formula (NUTRITION).

Hypothesis 1 examines whether a mother’s age (AGE), education (EDUCATION), income (INCOME), residence (LOCATION) and number of children (CHILDREN) influence her preference for advice from her close social circle over medical advice (MOMS_OVER_MED). The analysis revealed that when considering all independent variables together, the model does not perform significantly better than the null model (*p* = 0.187 > 0.05) (see Table A1 in Appendix A). However, when using AGE as a sole predictor, the model shows a significant improvement over the null model (*p* = 0.037 < 0.05) (see Table A2 in Appendix A). The Pearson and deviance statistics suggest that the model fits the data well, as both are not significant (*p* = 0.907 and *p* = 0.835, respectively) (see Table A3 in Appendix A). Finally, the likelihood ratio test table indicates that the variable AGE is significant at a 95% confidence level in this model (see Table A4 in Appendix A). The parameter estimates indicate that for every one-year increase in age, the odds of MOMS_OVER_MED being in category “0” (indifferent to the source of advice) versus category “1” (preferring close social circle over medical advice) increase by a factor of 1.048. This finding indicates a slight increase in the odds of preferring advice from the close social circle over medical advice as the mother’s age increases.

Hypothesis 2 examines whether a mother’s age (AGE), education (EDUCATION) and residence (LOCATION) affect her preference for advice from online social network forums over medical advice (NETWORKS_OVER_MED). The optimal model includes only the education level as a predictor. However, this model does not fit the data well, as it does not perform significantly better than the null model (*p* = 0.143 > 0.05) (see Table A5 in Appendix A), and the Goodness-of-Fit statistics indicate poor fit (*p* = 0.000 for both Pearson and deviance) (see Table A6 in Appendix A). Only the group of professional-certificate holders (EDUCATION = “2”) is significant for category “−1” which means that having a professional certificate education reduces the odds of preferring social network forums (category “−1”) by a factor of 0.525 compared to having an academic degree (category “3”) (see Table A7 in Appendix A).

Hypothesis 3 examines the factors influencing a mother’s preference for using infant formula over combining it with breastfeeding (MEAL). The optimal model is the one that includes the mother’s education level and the number of children as predictors. This model performs significantly better than the null model (*p* = 0.001 < 0.05) (see Table A8 in Appendix A), and the Goodness-of-Fit statistics indicate a good fit (*p* = 0.812 and *p* = 0.801 for Pearson and deviance, respectively) (see Table A9 in Appendix A). The parameter estimates reveal that Education = 1” is significant for category “0” (*p* = 0.024 < 0.05) (see Table A10 in Appendix A). Having up to a high school education increases the odds of preferring infant formula (category “0”) by a factor of 0.473 compared to having an academic degree (category “3”) (see Table A10 in Appendix A). EDUCATION = “2” is significant for category “0” (*p* = 0.000 < 0.05) (see Table A10 in Appendix A); therefore, having a professional certificate increases the odds of preferring infant formula (category “0”) by a factor of 0.379 compared to having an academic degree (category “3”). CHILDREN = “0” is significant for category “0” (*p* = 0.004 < 0.05) (see Table A10 in Appendix A); thereupon, having one child increases the odds of preferring infant formula (category “0”) by a factor of 0.547 in comparison to mothers with more than one child.

The results of Hypothesis 4 indicate that only education and the number of children are significant predictors for a feeding regimen that is not solely based on breastfeeding (NUTRITION). A refined model using these variables shows that EDUCATION = “1” (up to high school) increases the odds of NUTRITION being “1” (only infant formula) by a factor of 1.809 compared to academic-degree holders (EDUCATION = “3”) (see Table A11 in Appendix A). EDUCATION = “2” (professional certificate) increases the odds of NUTRITION being “1” (use of infant formula without breastfeeding) by a factor of 2.629 compared to academic-degree holders (EDUCATION = “3”) (see Table A11 in Appendix A). Having one child (CHILDREN = “0”) increases the odds of NUTRITION being “1” (use of infant formula without breastfeeding) by a factor of 1.454 compared to having more than one child (see Table A11 in Appendix A).

Analysis of Hypothesis 5 found a significant association between NUTRIRION and MEAL (*p* = 0.0001 < 0.05, Chi-Square statistic = 387.739). The variable MEAL is significant across all its categories. The odds ratio for MEAL = “0” (0–25% of the meals in the first three months are based on infant formula) is 0.003 (see Table A12 in Appendix A). Therefore, mothers that use infant formula in up to 25% of feedings in the first three months (MEAL = “0”) are less likely to base the meals exclusively on infant formula, compared to those who use infant formula for 75–100% of feedings in this period. Therefore, a higher percentage of meals with infant formula in the first three months correlates with a higher likelihood of the nutrition being exclusively infant formula.

However, no significant association was found between MEAL and MOMS_OVER_MED and between MEAL and NETWORKS_OVER_MED (*p* = 0.4867 > 0.05, Chi-Square statistic = 5.456; and *p* = 0.1688 > 0.05, Chi-Square statistic = 9.085, respectively).

Further examination reveals a statistically significant relationship between MOMS_OVER_MED and NETWORKS_OVER_MED (*p* = 0.007 < 0.05, Chi-Square statistic = 14.021). Subsequent multinomial logistic regression analysis reveals that mothers who prefer medical advice over social network recommendations (NETWORKS_OVER_MED = “−1”) are 2.098 times more likely to be indifferent about the advice either from their close social circle or medical professionals (MOMS_OVER_MED = “0”) (see Table A13 in Appendix A). Thus, mothers who prefer consulting with medical professionals have the least preference to seek advice from social networks.

## 5. Discussion

Our analysis reveals several important findings regarding the factors influencing the preferences of mothers seeking advice on the use of infant formula and feeding practices. First, the results suggest that age plays a significant role, as older mothers tend to prefer advice from their close social circle over medical professionals. This aligns with a previous study by Loudon et al. (2016) [37], who found that older mothers are more likely to seek curative care based on advice from their social circle rather than immediately consulting medical professionals. They attribute this insight to the findings of their study that indicate that the fear of judgment leads mothers to avoid healthcare professionals and to prefer the support of close social groups as a place to connect, seek and share information. Interestingly, our study found no significant effect of income level on a mother’s preference for social circle advice over medical advice, contrary to Loudon et al. (2016) [37], suggesting that socioeconomic status influences parenting decisions and access to resources. 

The observed relationship between preferences for advice from social circles and from social networks over medical advice is particularly intriguing. Mothers who prefer medical advice from social network recommendations are more likely to be indifferent about advice from their close social circle or medical professionals.

The education level and the number of children were found as significant variables that affect feeding regimens. Mothers with lower education levels (up to high school or professional certificate) were more likely to rely solely on infant formula compared to those with academic degrees. This finding enriches the conclusions of Moran-Lev et al. (2021) [16], by which mothers from lower socio-economic statuses preferred infant formula usage in comparison to mothers from higher socio-economic statuses. Nonetheless, Moran-Lev et al. (2021) [16] did not find a significant relation between the number of years of education of the parents and their feeding preferences.

Another significant finding is the exclusive use of infant formula among mothers with one child compared to those with multiple children. This finding suggests that experience and familiarity with infant feeding may influence the decision-making processes of new vs. experienced mothers. This result aligns with Kera et al. (2023) [38], who found that primiparity (having a first child) was significantly associated with a higher likelihood of formula feeding.

Our study also reveals a significant correlation between the percentage of meals with infant formula in the first three months and the likelihood of exclusively using infant formula. This finding highlights the importance of early feeding practices in shaping long-term infant nutrition patterns and the need for targeted support and education during this critical period. The finding is supported by the Academy of Breastfeeding Medicine’s protocol [39] that states that early supplementation with infant formula is associated with decreased exclusive breastfeeding rates in the first 6 months and an overall shorter duration of breastfeeding.

While Baker et al. (2021) [32] and Baker et al. (2023) [33] suggest that low socioeconomic status and poverty can influence parenting decisions and access to resources, no significant effect of income level on a mother’s tendency to rely more on her social circle’s advice versus medical professionals for child health issues was found. The differences in the findings of this study and prior studies can be explained by the setting of this research within a developed economy, while previous studies analyzed behavioral patterns within less-developed economies.

This complex interplay between different sources of advice reflects the multifaceted nature of decision-making in infant feeding and emphasizes the need for a comprehensive approach to supporting new mothers. In particular, these findings have important implications for healthcare providers, policymakers and those developing support systems for new mothers. They suggest a need for tailored approaches that consider individual circumstances and preferences when providing information and support on infant feeding. From a health policy perspective, the significant impact of education level on feeding choices highlights the importance of accessible, comprehensive education on infant nutrition for all mothers, regardless of their educational background. Further, the correlation between early formula use and long-term feeding patterns emphasizes the critical nature of support and guidance in the first months of an infant’s life. In particular, following the observation that the exclusive use of infant formula affects the later administration of baby-feeding, hospitals, healthcare institutes and public health organizations play a major role in enhancing both the exclusivity and the duration of breastfeeding.

The results highlight the potential influence of social networks and online forums on mothers’ decision-making processes, emphasizing the need for reliable, evidence-based information in this domain. In light of the influence of social networks and online forums, utilizing these platforms to provide accurate, evidence-based information to support informed decision-making has major potential to contribute to improved health outcomes for both mothers and infants.

## 6. Conclusions

Our study contributes to understanding the complex relations between various demographic and socioeconomic factors and the preferences and choices of mothers regarding the use of infant formula and how they are affected by their social circle and social networks. Our findings demonstrate that a mother’s age, education level and number of children play significant roles in shaping these preferences and choices. The complex relationships observed between different sources of advice and feeding practices highlight the multifaceted nature of decision-making in infant nutrition.

The observed relationship between the preference for advice from social circles and social networks over medical advice is particularly intriguing. Mothers who prefer medical advice over social network recommendations are more likely to exhibit indifference towards advice from both their close social circle and medical professionals. The Remedia case discussed in this paper highlights the notion that implementing strict regulations on the composition of infant formulas can provide enhanced protection for children who, due to various circumstances, do not receive nutrition that is based on breastfeeding.

Future research should further explore the role of cultural factors and their impact on the dynamic relationships between different sources of advice and infant feeding practices. Moreover, studies examining the influence of close social circles, medical professionals and social networks in both developed and less-developed countries can provide useful insights into early feeding decisions, supporting health policies worldwide.

## Data Availability

Data is available from the authors upon request.

## Appendix A

**Table A1:** Model fitting information for Hypothesis 1.

Model Fitting Criteria				Likelihood Ratio Tests		
Model	AIC	BIC	−2 Log Likelihood	Chi-Square	df	Sig.
Intercept Only	690.902	699.712	686.902			
Final	700.445	770.929	668.445	18.457	14	0.187

**Table A2:** Likelihood ratio tests for Hypothesis 1.

Model Fitting Criteria				Likelihood Ratio Tests		
Effect	AIC of Reduced Model	BIC of Reduced Model	−2 Log Likelihood of Reduced Model	Chi-Square	df	Sig.
Intercept	197.041	205.851	193.041	23.132	2	0.000
Age	180.527	189.338	176.527	6618	2	0.037

**Table A3:** Goodness-of-Fit for Hypothesis 1.

	Chi-Square	df	Sig.
Pearson	197.041	205.851	193.041
Deviance	180.527	189.338	176.527

**Table A4:** Parameter estimates for Hypothesis 1.

					95% Confidence Interval for Exp(B)			
**Moms_Over_Med**	**B**	**Std. Error**	**Wald**	**df**	**Sig.**	**Exp(B)**	**Lower Bound**	**Upper Bound**
−1.0 Intercept	−3.022	0.913	10.948	1	0.001			
Age	0.037	0.027	1.869	1	0.172	1.038	0.984	1.094
0.0 Intercept	−2.521	0.668	14.253	1	0.000			
Age	0.046	0.020	5.505	1	0.019	1.048	1.008	1.089

**Table A5:** Model fitting information for Hypothesis 2.

Model Fitting Criteria				Likelihood Ratio Tests		
Model	AIC	BIC	−2 Log Likelihood	Chi-Square	df	Sig.
Intercept Only	39.769	48.579	35.769			
Final	40.899	67.330	28.899	6.870	4	0.143

**Table A6:** Goodness-of-Fit for Hypothesis 2.

	Chi-Square	df	Sig.
Pearson	0.000	0	
Deviance	0.000	0	

**Table A7:** Parameter estimates for Hypothesis 2.

					95% Confidence Interval for Exp(B)			
**Networks _Over_Med**	**B**	**Std. Error**	**Wald**	**df**	**Sig.**	**Exp(B)**	**Lower Bound**	**Upper Bound**
−1 Intercept	1.476	0.142	108.233	1	0.000			
[Education = 1]	0.037	0.027	1.869	1	0.172	1.038	0.984	1.094
[Education = 2]	−0.643	0.303	4.507	1	0.034	0.525	0.290	0.952
[Education = 3]	0			0				
0 Intercept	0.779	0.155	25.409	1	0.000			
[Education = 1]	−0.086	0.523	0.027	1	0.869	0.917	0.329	2.559
[Education = 2]	−0.640	0.343	3.486	1	0.062	0.527	0.269	1.032
[Education = 3]	0			0				

**Table A8:** Model fitting information for Hypothesis 3.

Model Fitting Criteria				Likelihood Ratio Tests		
Model	AIC	BIC	−2 Log Likelihood	Chi-Square	df	Sig.
Intercept Only	104.431	117.647	98.431			
Final	95.071	147.934	71.071	27.361	9	0.001

**Table A9:** Goodness-of-Fit for Hypothesis 3.

	Chi-Square	df	Sig.
Pearson	2.974	6	0.812
Deviance	3.063	6	0.801

**Table A10:** Parameter estimates for Hypothesis 3.

					95% Confidence Interval for Exp(B)			
**Meal**	**B**	**Std. Error**	**Wald**	**df**	**Sig.**	**Exp(B)**	**Lower Bound**	**Upper Bound**
0 Intercept	0.925	0.136	46.462	1	0.000			
[Education = 1]	−0.749	0.331	5.131	1	0.024	0.473	0.247	0.904
[Education = 2]	−0.971	0.271	12.886	1	0.000	0.379	0.223	0.643
[Education = 3]	0			0				
[Children = 0]	−0.603	0.208	8.411	1	0.004	0.547	0.364	0.822
[Children = 1]	0			0				
1 Intercept	−0.910	0.214	18.112	1	0.000			
[Education = 1]	−0.295	0.495	0.354	1	0.552	0.745	0.282	1.964
[Education = 2]	−0.727	0.452	2.590	1	0.108	0.483	0.199	1.172
[Education = 3]	0			0				
[Children = 0]	−0.142	0.317	0.200	1	0.655	0.868	0.466	1.616
[Children = 1]	0			0				
2 Intercept	−1.004	0.217	21.421	1	0.000			
[Education = 1]	−0.144	0.470	0.094	1	0.759	0.866	0.345	2.173
[Education = 2]	−0.071	0.371	0.037	1	0.848	0.931	0.450	1.928
[Education = 3]	0			0				
[Children = 0]	0.134	0.298	0.201	1	0.654	1.143	0.637	2.050
[Children = 1]	0			0				

**Table A11:** Variables in the equation for Hypothesis 4.

	B	S.E	Wald	df	Sig.	Exp(B)
Education			18.422	2	0.000	
Education (1)	0.593	0.291	4.134	1	0.042	1.809
Education (2)	0.967	0.239	16.395	1	0.000	2.629
Children (1)	0.374	0.186	4.049	1	0.044	1.454
Constant	−0.966	0.124	60.945	1	0.000	0.381

**Table A12:** Variables in the equation for Hypothesis 5.

	B	S.E	Wald	df	Sig.	Exp(B)
Meal			176.416	3	0.000	
Meal (1)	−5.943	0.502	140.426	1	0.000	0.003
Meal (2)	−3.580	0.423	71.515	1	0.000	0.028
Meal (3)	−1.410	0.333	17.913	1	0.000	0.244
Constant	1.865	0.219	72.314	1	0.000	6.458

**Table A13:** Parameter estimates for Hypothesis 5.

					95% Confidence Interval for Exp(B)			
**Moms_Over_Med**	**B**	**Std. Error**	**Wald**	**df**	**Sig.**	**Exp(B)**	**Lower Bound**	**Upper Bound**
−1 Intercept	−2.244	0.398	31.861	1	0.000			
[Networks_Over_Med = −1]	0.773	0.429	3.251	1	0.071	2.165	0.935	5.015
[Networks_Over_Med = 0]	−0.224	0.516	0.189	1	0.664	0.799	0.291	2.198
[Networks_Over_Med = 1]	0			0				
0 Intercept	−1.551	0.294	27.770	1	0.000			
[Networks_Over_Med = −1]	0.741	0.320	5.375	1	0.020	2.098	1.121	3.924
[Networks_Over_Med = 0]	0.469	0.346	1.831	1	0.176	1.598	0.810	3.152
[Networks_Over_Med = 1]	0			0				
